# Urinary Proteins, Vitamin D and Genetic Polymorphisms as Risk Factors for Febrile Urinary Tract Infection and Relation with Bacteremia: A Case Control Study

**DOI:** 10.1371/journal.pone.0121302

**Published:** 2015-03-25

**Authors:** Willize E. van der Starre, Cees van Nieuwkoop, Uginia Thomson, Marleen S. M. Zijderveld-Voshart, Jan Pieter R. Koopman, Tanny J. K. van der Reijden, Jaap T. van Dissel, Esther van de Vosse

**Affiliations:** 1 Department of Infectious Diseases, Leiden University Medical Center, Leiden, The Netherlands; 2 Department of Internal Medicine, Haga Hospital, the Hague, The Netherlands; IIBB-CSIC-IDIBAPS, SPAIN

## Abstract

**Objective/Purpose:**

Febrile urinary tract infection (UTI) is a common bacterial disease that may lead to substantial morbidity and mortality especially among the elderly. Little is known about biomarkers that predict a complicated course. Our aim was to determine the role of certain urinary cytokines or antimicrobial proteins, plasma vitamin D level, and genetic variation in host defense of febrile UTI and its relation with bacteremia.

**Methods:**

A case-control study. Out of a cohort of consecutive adults with febrile UTI (n = 787) included in a multi-center observational cohort study, 46 cases with bacteremic *E*.*coli* UTI and 45 cases with non-bacteremic *E*.*coli* UTI were randomly selected and compared to 46 controls. Urinary IL-6, IL-8, LL37, β-defensin 2 and uromodulin as well as plasma 25-hydroxyvitamin D were measured. In 440 controls and 707 UTI patients polymorphisms were genotyped in the genes *CXCR1*, *DEFA4*, *DEFB1*, *IL6*, *IL8*, *MYD88*, *UMOD*, *TIRAP*, *TLR1*, *TLR2*, *TLR5* and *TNF*.

**Results:**

IL-6, IL-8, and LL37 are different between controls and UTI patients, although these proteins do not distinguish between patients with and without bacteremia. While uromodulin did not differ between groups, inability to produce uromodulin is more common in patients with bacteremia. Most participants in the study, including the controls, had insufficient vitamin D and, at least in winter, UTI patients have lower vitamin D than controls. Associations were found between the CC genotype of *IL6* SNP rs1800795 and occurrence of bacteremia and between *TLR5* SNP rs5744168 and protection from UTI. The rare GG genotype of *IL6* SNP rs1800795 was associated with higher β-defensin 2 production.

**Conclusion:**

Although no biomarker was able to distinguish between UTI with or without bacteremia, two risk factors for bacteremia were identified. These were inability to produce uromodulin and an *IL6* rs1800795 genotype.

## Introduction

Urinary tract infection (UTI) is one of the most common bacterial infections and has a high risk of recurrence. The predominant causal pathogen is uropathogenic *Escherichia coli* and it is thought that innate immune responses to this pathogen can both control and predispose to subsequent recurrence of UTI [1]. Although UTI by itself rarely causes significant complications, fever indicates the presence of tissue inflammation that can be accompanied with bacteremia and the urosepsis syndrome that may eventually lead to septic shock and death [2].

We have set up a large prospective multicenter cohort study to investigate various aspects of febrile UTI [3–5]. In this study we focus on urinary markers of host defense, plasma vitamin D and the role of genetic variation.

The cytokines IL-6 and IL-8 are mediators of inflammation in response to bacterial infection, and when measured in plasma or serum these may be used as early biomarkers of infection. IL-6 and IL-8 levels are also known to be elevated in the urine of patients with UTI, whereas reportedly none are measurable in the urine of healthy controls [6–10].

The human body produces several antimicrobial proteins, amongst others cathelicidin LL37 and β-defensins, that are part of our first line defense against bacterial infections. These antimicrobial proteins are produced by epithelial cells, including those of the urinary tract [11,12]. Urinary cathelicidin is elevated in children with pyelonephritis [11] and was recently reported to be also elevated in adult women during UTI [13]. Whether the amounts of cathelicidin or β-defensin produced in the urine of UTI patients is associated with disease severity such as bacteremia is unknown.

Uromodulin (also known as Tamm Horsfall protein) is the most abundant urinary protein in mammals. It adheres to fimbriae of uropathogenic *E*. *coli*, thus preventing its attachment to the epithelium. Uromodulin also has an immunomodulatory function [14]. In young women with recurrent UTI urinary uromodulin concentrations were similar to those in healthy controls [15]. Whether uromodulin lowers the risk for developing febrile UTI or associated bacteremia is unknown.

Vitamin D is known to play an important role in the first defense against bacterial infections; e.g. by induction of the antimicrobial proteins cathelicidin and β-defensin. High prevalence of vitamin D deficiency and insufficiency have been reported in elderly people (≥ 65 years) in the Netherlands [16,17] just as in many other regions around the world [18]. It has been shown in *in vitro* experiments that bladder epithelium from women taking vitamin D supplements are capable of producing larger amounts of cathelicidin upon infection [19]. Thus vitamin D deficiency is believed to be an attributable factor in host susceptibility to UTI as has also been suggested in a recent case-control study [20]. Whether vitamin D status of UTI patients affects cathelicidin and β-defensin production in urine and the occurrence of bacteremia is unknown.

Many studies have found that host genetic factors influence susceptibility to human infectious diseases [21,22]. Also in UTI genetic factors likely play a role, as illustrated by the finding that positive family history is a risk factor for recurrent UTI [23] and reviewed in [1]. Studies evaluating the role of genetic factors in UTI were mainly performed in children [24–29]. No studies have investigated the contribution of host genetic factors to the development of UTI complicated by bacteremia.

The aims of the current study were: first, to investigate the production of cytokines and antimicrobial proteins in the urine of UTI patients in order to determine whether any of these proteins are risk factors for febrile UTI or predictive biomarkers for bacteremia; second, to determine whether plasma vitamin D is correlated with urinary protein production or clinical outcome; and third, to determine whether there is a correlation between genetic variants and the production of any of the proteins or occurrence of bacteremia.

## Materials and Methods

### Patients and controls

**Fig 1:**
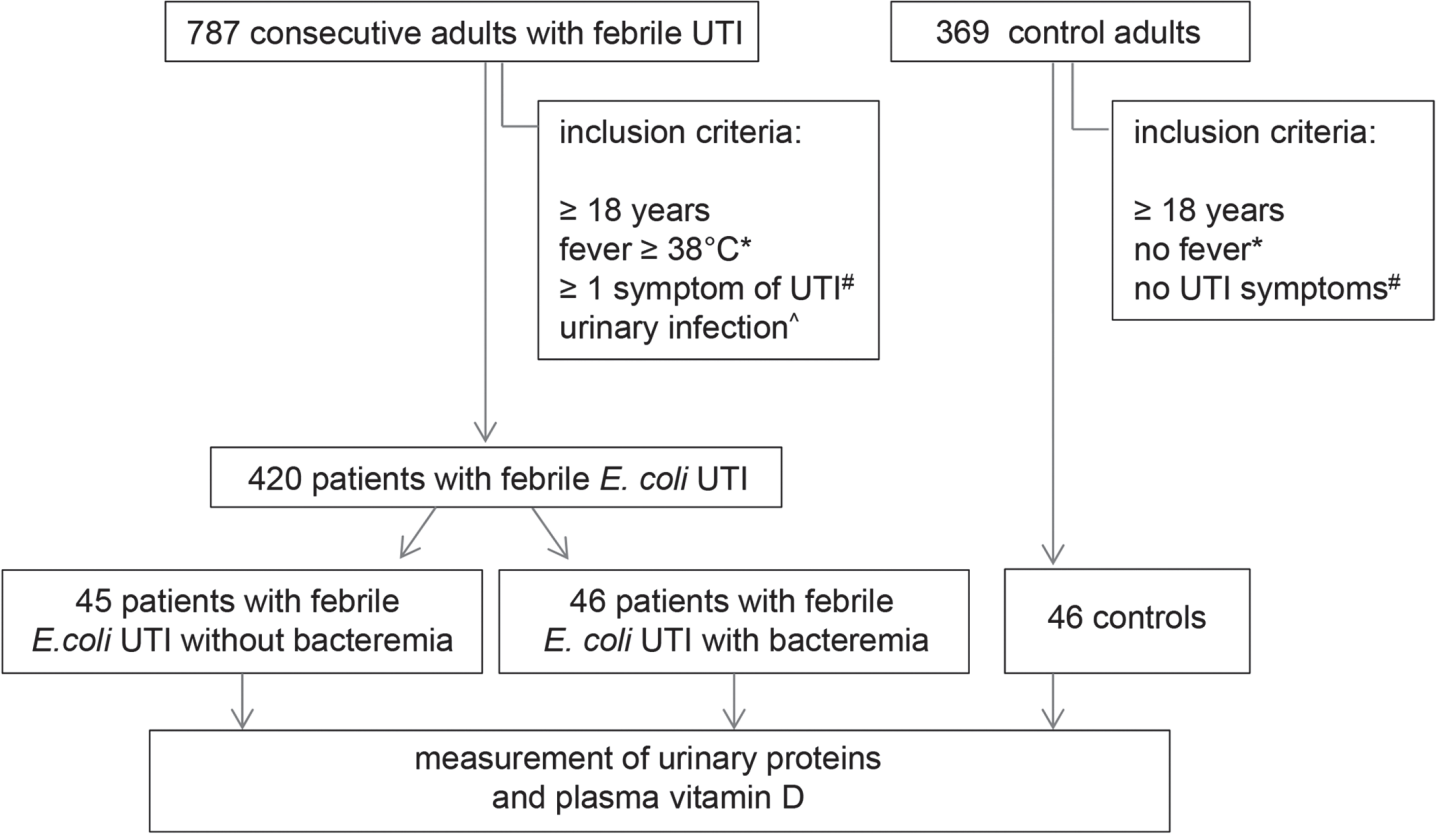
Selection of UTI patients and controls for the urine and plasma measurements. *and/or a history of fever and chills within 24 hours before presentation. #Symptoms of UTI: dysuria, increased urination frequency, urgency, perineal pain, flank pain or costovertebral tenderness. ^Urinary infection as determined by a positive nitrite dipstick test or leukocyturia as defined by a positive leukocyte esterase dipstick test or the presence of more than five leukocytes per high-power field (pyuria) in a centrifuged sediment.

We conducted a prospective observational multi-center cohort study. Patients were recruited from 35 family practices and emergency departments of eight hospitals in the region. Consecutive patients (n = 787) with a presumptive diagnosis of febrile UTI were considered for enrollment and those who met the entry criteria and provided written informed consent were included. Inclusion criteria for the patients were: age ≥18 years, fever (≥38.0°C) and/or a history of fever and chills within 24 hours before presentation, at least one symptom of UTI (dysuria, increased urination frequency, urgency, perineal pain, flank pain or costovertebral tenderness) and a positive nitrite dipstick test or leukocyturia as defined by a positive leukocyte esterase dipstick test or the presence of more than five leukocytes per high-power field (pyuria) in a centrifuged sediment. Exclusion criteria were: pregnancy, hemodialysis or peritoneal dialysis, a history of kidney transplantation, known presence of polycystic kidney disease or current treatment for urolithiasis or hydronephrosis. From the patients with *E*. *coli* as the causal uropathogen 45 patients without bacteremia and 46 with bacteremia were randomly selected for the plasma and urine measurements (**Fig. 1**). Controls (n = 369) were recruited from the same primary healthcare centers and emergency departments if they were aged ≥18 years, had no symptoms of UTI, and provided written informed consent. Of these controls included from the primary health care centers, 46 non-febrile individuals were randomly selected for the plasma and urine measurements (**Fig. 1**). The study was approved by the Medical Ethics Committee of the Leiden University Medical Center (approval number P08.065). Blood and urine samples were taken before the start of antimicrobial treatment. Plasma and urine were stored at -80°C within two hours after enrollment. Blood and urine cultures were analyzed using standard microbiological methods. DNA was isolated from white blood cells by a salting out method essentially as described [30] and stored at -20°C.

### Measuring proteins in urine

Protein specific enzyme-linked immunosorbent assays (ELISA) were used to determine concentrations in urine essentially according the manufacturer's protocol with minor modifications. Urine samples were diluted two and ten times for the IL-6 ELISA, eight and 64 times for the IL-8 ELISA (Biosource). The detection limit of both ELISAs was 30 pg/ml. Urine samples were diluted four and 32 times for the LL37 ELISA (Hycult Biotechnology), the detection limit was 2 ng/ml. Urine samples were diluted four and 32 times for the β-defensin 2 ELISA (Antigenix America Inc), the detection limit was 10 pg/ml. Urine samples were diluted 200 and 400 times for the Uromodulin ELISA (MD Bioproducts), the detection limit was 20 ng/ml. Optical densities were determined at 450 nm in an iMark microplate reader (Bio-Rad).

In order to account for the gravity of the urine, protein concentrations were divided by the urine creatinine concentration. Urinary concentrations of creatinine were measured with the CREA plus (Cobas) enzymatic assay in an automated clinical chemistry analyzer.

### Vitamin D assay

The concentration of 25-hydroxyvitamin D (25(OH)D) was determined in plasma by a competitive electrochemiluminescence immunoassay (ECLIA), essentially according to the manufacturer's protocol (Elecsys Vitamin D Total assay, Roche Diagnostics). Based on consensus in the literature [31] we defined vitamin D deficiency as a plasma 25(OH)D concentration ≤20 ng/ml, relative vitamin D insufficiency at plasma 25(OH)D concentrations between 20 and 30 ng/ml, and all concentrations ≥30 ng/ml as sufficient.

### Genotyping

Of the 787 enrolled patients, 707 were included in the genotyping assays (of 80 samples DNA was not isolated or of low quality), as well as 440 controls (369 controls from the cohort study as well as anonymous controls). Genotyping of polymorphisms was performed by use of a Sequenom MassArray platform according to the manufacturer’s protocols (Sequenom, San Diego, USA). Multiplex assays were designed with Assay designer software (Sequenom). In brief, after a multiplex PCR on 5 ng of DNA, a primer extension reaction was performed to introduce mass-differences between alleles and, after removing salts by adding a resin, 15 nl of the product was spotted onto a target chip with 384 patches containing matrix. Mass differences were detected using a Bruker Autoflex MALDI-TOF mass spectrometer and genotypes were assigned real-time using Typer 3.1 software (Sequenom). Several samples representing the various genotypes were sequenced to confirm the genotyping results. As quality control, 10% of samples were genotyped in duplo; no inconsistencies were observed. Primer and probe sequences can be found in Table 1.

**Table 1:** Primer and probe sequences for the multiplex genotyping assay.

gene	SNPid	F primer sequence	R primer sequence	Probe sequence
*TNF*	rs1800629	ACGTTGGATGGGAGGCAATAGGTTTTGAGG	ACGTTGGATGTTCTGGGCCACTGACTGATT	GGCTGAACCCCGTCC
*DEFB1*	rs1800972	ACGTTGGATGCTGTCAGCTCAGCCTCCAAA	ACGTTGGATGTCATGGCGACTGGCAGGCAA	CAGGAACTGGGGAGA
*UMOD*	rs4293393	ACGTTGGATGGCTGAGAATGGCTGAAAGTC	ACGTTGGATGTGTTGTACAGAGTGGGTCAG	CAGGTCCAGTGATGTC
*TIRAP*	rs8177374	ACGTTGGATGGTACATGAATCGGAGCTCAG	ACGTTGGATGGCCGAGGGCTGCACCATCC	CACCATCCCCCTGCTGT
*IL6*	rs1800795	ACGTTGGATGAGCCTCAATGACGACCTAAG	ACGTTGGATGGATTGTGCAATGTGACGTCC	GTGACGTCCTTTAGCAT
*TLR1*	rs5743618	ACGTTGGATGTAACTCTGCTGATCGTCACC	ACGTTGGATGTGAGATACCAGGGCAGATCC	CAGGGCAGATCCAAGTAG
*CXCR1*	rs2234671	ACGTTGGATGGAATCTCAGTGGCATCCAGG	ACGTTGGATGTGAGGACCCAGGTGATCC	ggCAGGTGATCCAGGAGA
*DEFA4*	rs28661751	ACGTTGGATGATCACCTCTTGCCTGGAGTG	ACGTTGGATGCAGCCATGAGGATTATCGCC	tAGGATTATCGCCCTCCTC
*TLR2*	rs5743708	ACGTTGGATGAAAAAAGCCATTCCCCAGCG	ACGTTGGATGCAGGTAGGTCTTGGTGTTCA	TCTTGGTGTTCATTATCTTC
*TLR5*	rs5744168	ACGTTGGATGTCCTGGAAAAATTACAGACC	ACGTTGGATGAGATATCGGGTATGCTTGG	GGTTGTAAGAGCATTGTCTC
*MYD88*	rs6853	ACGTTGGATGGCGTACAAAACATGTAGAAG	ACGTTGGATGCACCTGTCCCCCTTTAATAC	ccGGCATTTTAAAGCCATCTC
*DEFA4*	rs736227	ACGTTGGATGGTTCCCAGCATGACATTCTC	ACGTTGGATGGTTTCACATACTGCTGCACG	ggtgaACGCGTGTCGATTAAC
*CXCR1*	rs3138060	ACGTTGGATGTGTGGGAGCTGAGGATTTCT	ACGTTGGATGTCCTCTTCACCTGCTAACTC	CTGCTAACTCCATGTATGAGTG
*IL8*	rs4073	ACGTTGGATGGGTACTATGATAAAGTTATCT	ACGTTGGATGCTGAAGCTCCACAATTTGGT	CTCCACAATTTGGTGAATTATCAA
*IL6*	rs10499563	ACGTTGGATGAAGCCTGGTCTGGCCTGTAT	ACGTTGGATGACCTGAAAGGAGGTAGCAGA	GATTTCTTAATTATTATACAAGCACA

### Statistical analyses

All data were entered in an SPSS database (SPSS Inc., Chicago, IL, USA; version 20.0) for statistical analysis. Graphs were generated using GraphPad Prism (GraphPad Software Inc., San Diego, California, USA; version 5.01). Descriptive analysis included medians and ranges, or means or percentages with standard deviation (sd), as appropriate. Univariate analysis was performed using the Student’s t–test, the Kruskal-Wallis test or the Mann–Whitney U-test for continuous variables and the Pearson Chi-Square test for categorical variables. Measures for association were expressed as odds ratios (ORs) with their 95% confidence intervals (CI) for categorical variables.

## Results

### Description of patients and controls for urine and plasma measurements

During the study period, 787 patients with febrile UTI were enrolled. *E*. *coli* was the most frequent causal uropathogen, present in 420 (53%) of the patients. From these, 45 patients without bacteremia and 46 patients with bacteremia were selected and compared to 46 controls (**Fig. 1**). Controls had a median age (58 years, IQR 49–75) comparable to UTI patients without bacteremia (63 years, IQR 46–75), but significant lower than UTI patients with bacteremia (73 years, IQR 60–82). The groups were comparable with respect to sex, BMI, use of immunosuppressants and vitamin D supplements. Further baseline characteristics of the study population are summarized in **Table 2**.

**Table 2:** Baseline characteristics of the study population.

	controls,	febrile UTI	febrile UTI
		without bacteremia,	with bacteremia,
	*n* = 46	*n* = 45	*n* = 46
Age in years, median [IQR]	58 [49–75]	63 [46–75]	73 [60–82]
Male sex	18 (39)	16 (36)	18 (39)
**Comorbidity**			
Any	24 (52)	33 (73)	36 (78)
Urinary tract disorder ^a^	5 (11)	10 (22)	9 (20)
Urinary catheter	0 (0)	2 (4)	3 (7)
History of nephrolithiasis	5 (11)	4 (9)	5 (11)
Recurrent UTIs ^b^	1 (2)	16 (36)	6 (13)
Diabetes mellitus	3 (7)	5 (11)	12 (26)
Malignancy	2 (4)	2 (4)	4 (9)
Hypertension	17 (37)	24 (53)	26 (57)
Heart failure	0 (0)	5 (11)	8 (17)
Cerebrovascular disease	1 (2)	5 (11)	12 (26)
Chronic renal insufficiency	0 (0)	3 (7)	6 (13)
COPD	2 (4)	7 (16)	3 (7)
**Medication**			
Immunosuppressants	1 (2)	1 (2)	4 (9)
Vitamin D supplements	2 (4)	4 (9)	4 (9)
**Season of inclusion**			
Winter (Dec—Feb)	46 (100)	8 (18)	5 (11)
Spring (Mar—May)	-	6 (13)	10 (22)
Summer (June—Aug)	-	11 (24)	17 (37)
Fall (Sept—Nov)	-	20 (44)	14 (30)
**BMI** ^c^			
Mean BMI in kg/m^2^ (sd)	26.5 (4.6)	26.6 (4.0)	27.3 (7.2)

Data are presented as n (%) unless otherwise stated. UTI = urinary tract infection, IQR = interquartile range, sd = standard deviation, COPD = chronic obstructive pulmonary disease, BMI = body mass index. ^a^ Defined as any functional or anatomical abnormality of the urinary tract except urinary catheter and history of nephrolithiasis. ^b^ Defined as ≥2 UTIs in the last 6 months or ≥3 UTIs in the last 12 months. ^c^ Eight missing BMI data: 2 in controls, 1 in UTI without bacteremia and 5 in UTI with bacteremia.

### Cytokines in urine do not distinguish between patients with our without bacteremia

**Fig 2:**
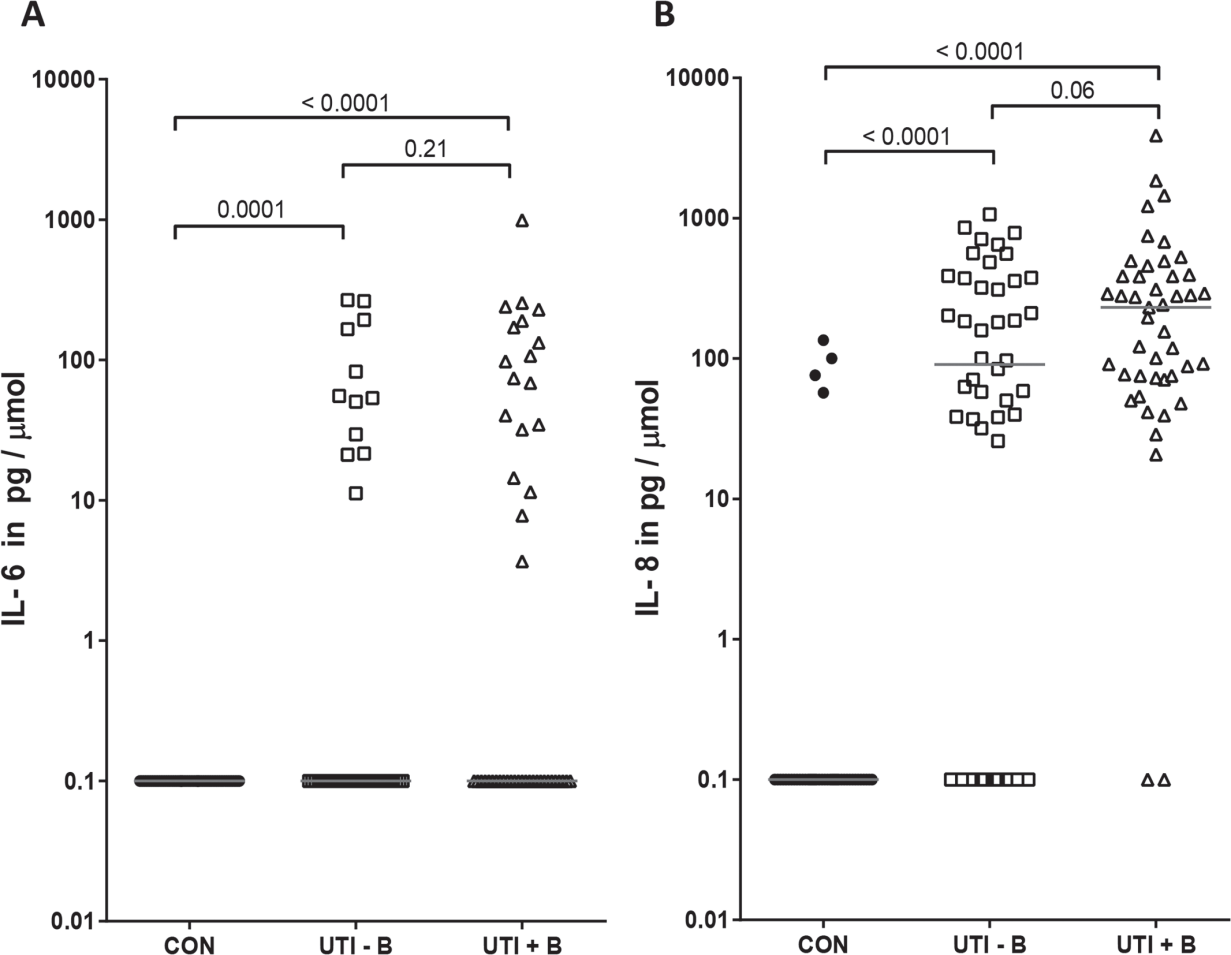
IL-6 or IL-8 concentrations do not distinguish UTI patients with bacteremia from those without bacteremia. IL-6 (**A**) and IL-8 (**B**) concentrations were determined in the urine of 46 controls (CON), 44 cases with febrile UTI without bacteremia (UTI–B), and 45 cases with febrile UTI with bacteremia (UTI+B). Cytokine concentrations depicted were corrected for urine gravity, for samples below the detection limit the corrected cytokine concentration was set to 0.1. Solid bars represent medians, for statistical analysis a Mann-Whitney test was used.

The production of the cytokines IL-6 and IL-8 was determined in urine samples from 44 UTI patients without bacteremia, 45 UTI patients with bacteremia and 46 controls. IL-6 was detected in 30 of all 89 (34%) UTI patients, in 12 of 44 (27%) without bacteremia and in 18 of 45 (40%) with bacteremia, and in none of the controls. Due to the large number of samples with undetectable IL-6 in each group, the median IL-6 concentrations were the same in the three groups. Both patient groups are significantly different from the controls (each p<0.001) but not significantly different from each other (p = 0.21) (**Fig. 2A**).

IL-8 was detected in 77 of all 89 (87%) UTI patients, in 34 of 44 (77%) without bacteremia and in 43 of 45 (96%) with bacteremia, and in only four of the 46 controls (9%). The median value of the controls is 0.1 pg IL-8 /μmol creatinine, of the UTI patients without bacteremia 91 pg/μmol and of those with bacteremia 232 pg/μmol. Both patient groups are significantly different from the controls (each p<0.0001) but not significantly different from each other (p = 0.06) (**Fig. 2B**).

A positive correlation was demonstrated between IL-8 production and age (r = 0.302, p<0.001), IL-6 production (r = 0.534, p<0.001), and temperature at onset (r = 0.516, p<0.001). IL-8 was not correlated with presence of urinary tract disorders, recurrent UTIs or antibiotic pre-treatment.

### Antimicrobial proteins in urine do not correlate with bacteremia

**Fig 3:**
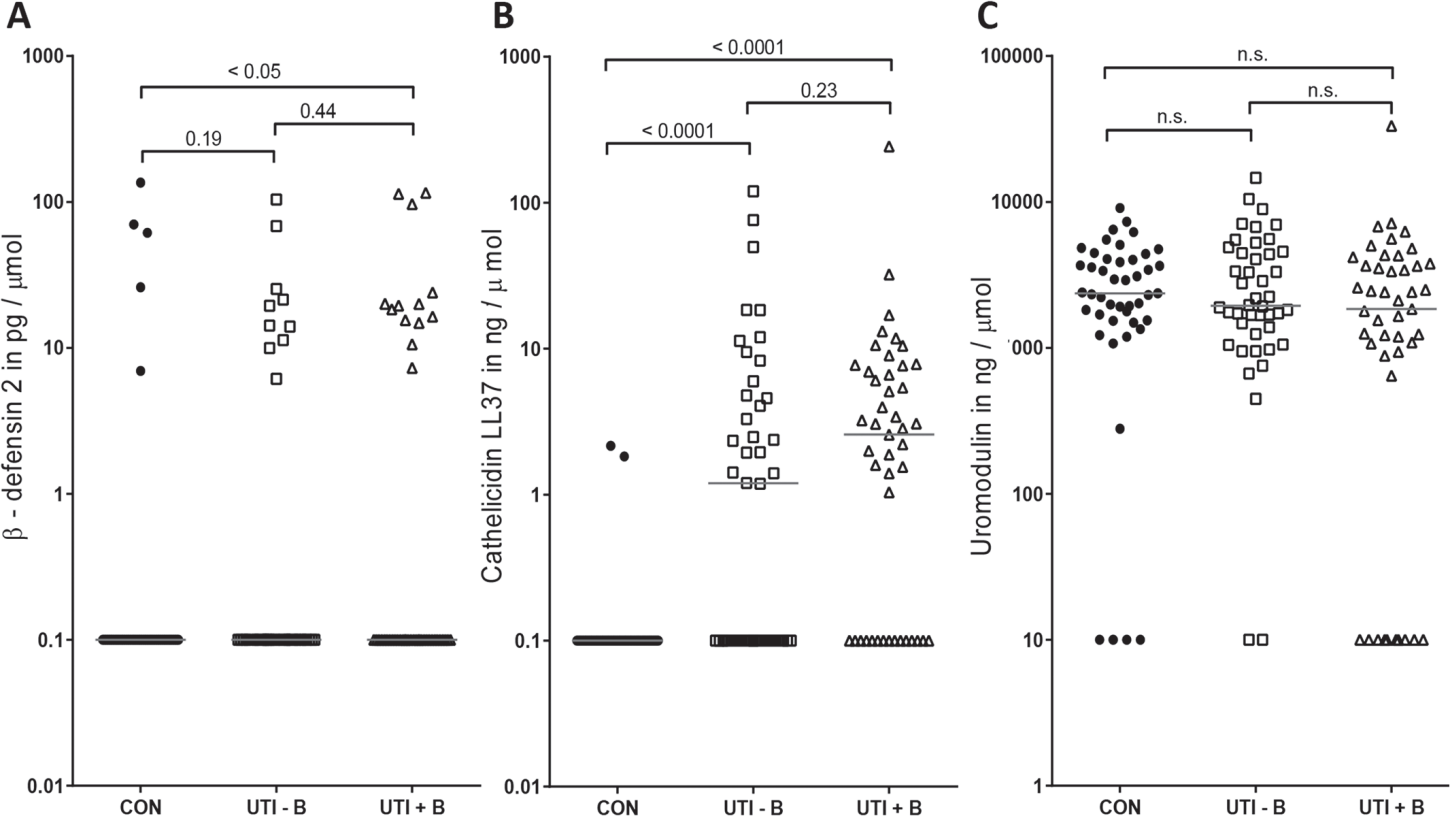
Antimicrobial protein concentrations do not distinguish UTI patients with bacteremia from those without bacteremia. Concentrations of the antimicrobial proteins β-defensin 2 (**A**) Cathelicidin LL37 (**B**) and Uromodulin (**C**) were determined in the urine of 46 controls (CON), 44 febrile UTI cases without bacteremia (UTI–B), and 45 febrile UTI cases with bacteremia (UTI+B). Protein concentrations depicted were corrected for urine gravity, for samples below the detection limit the corrected protein concentration was set to 0.1 (β-defensin 2, Cathelicidin LL37) or 10 (Uromodulin). Solid bars represent medians, for statistical analysis the Mann-Whitney U test was used.

The antimicrobial protein β-defensin 2 was detected in 23 of all 89 (26%) UTI patients, in 10 of 44 (23%) with bacteremia and in 13 of 45 (29%) without bacteremia, and in five of the 46 controls (11%). Due to the large number of samples with undetectable β-defensin 2 in each group, the median concentrations were similar in the three groups. Only the UTI patient group with bacteremia was significantly different from the controls (p<0.05) (**Fig. 3A**).

Urinary β-defensin 2 showed no correlation with age, IL-8 production and temperature at onset. The β-defensin 2 was also not correlated with presence of urinary tract disorders, recurrent UTIs or antibiotic pre-treatment.

Cathelicidin LL37 was detectable in 53 of all 89 (60%) UTI patients, in 23 of 44 (52%) without bacteremia and in 30 of 45 (67%) with bacteremia, and in two of the 46 controls (4%). The median value of the controls is below the detection limit, of the UTI patients without bacteremia it is 1.2 ng LL37 /μmol creatinine and of those with bacteremia 2.6 ng/μmol. Both patient groups are significantly different from the controls (each p<0.0001) but not significantly different from each other (p = 0.23) (**Fig. 3B**). LL37 showed no difference in the presence of urinary tract disorders, recurrent UTIs and antibiotic pretreatment.

### Lack of uromodulin in urine increases risk of bacteremia

Uromodulin was detectable in 78 of all 89 (88%) UTI patients, in 42 of 44 (95%) without bacteremia and in 35 of 45 (78%) with bacteremia, and in 42 of the 46 (91%) controls. The median value of the controls is 2366 ng uromodulin /μmol creatinine, of the UTI patients without bacteremia it is 1941 ng/μmol and of those with bacteremia 1851 ng/μmol. The uromodulin production is not significantly different between the groups (**Fig. 3C**).

Most people produce uromodulin in the urine regardless of infection and it appears that inability to produce any uromodulin is more common in the patients with bacteremia than in patients without bacteremia (p = 0.015). The odds ratio for developing bacteremia for UTI patients who do not produce uromodulin is 6.0 (95% CI: 1.2–29.2).

Uromodulin showed no correlation with blood leukocyte count, age, IL-8 or temperature at onset. Also, uromodulin did not differ in the presence of leukocyturia, urinary tract disorders, recurrent UTIs or antibiotic pre-treatment.

### Most participants in the study had insufficient vitamin D levels

**Fig 4:**
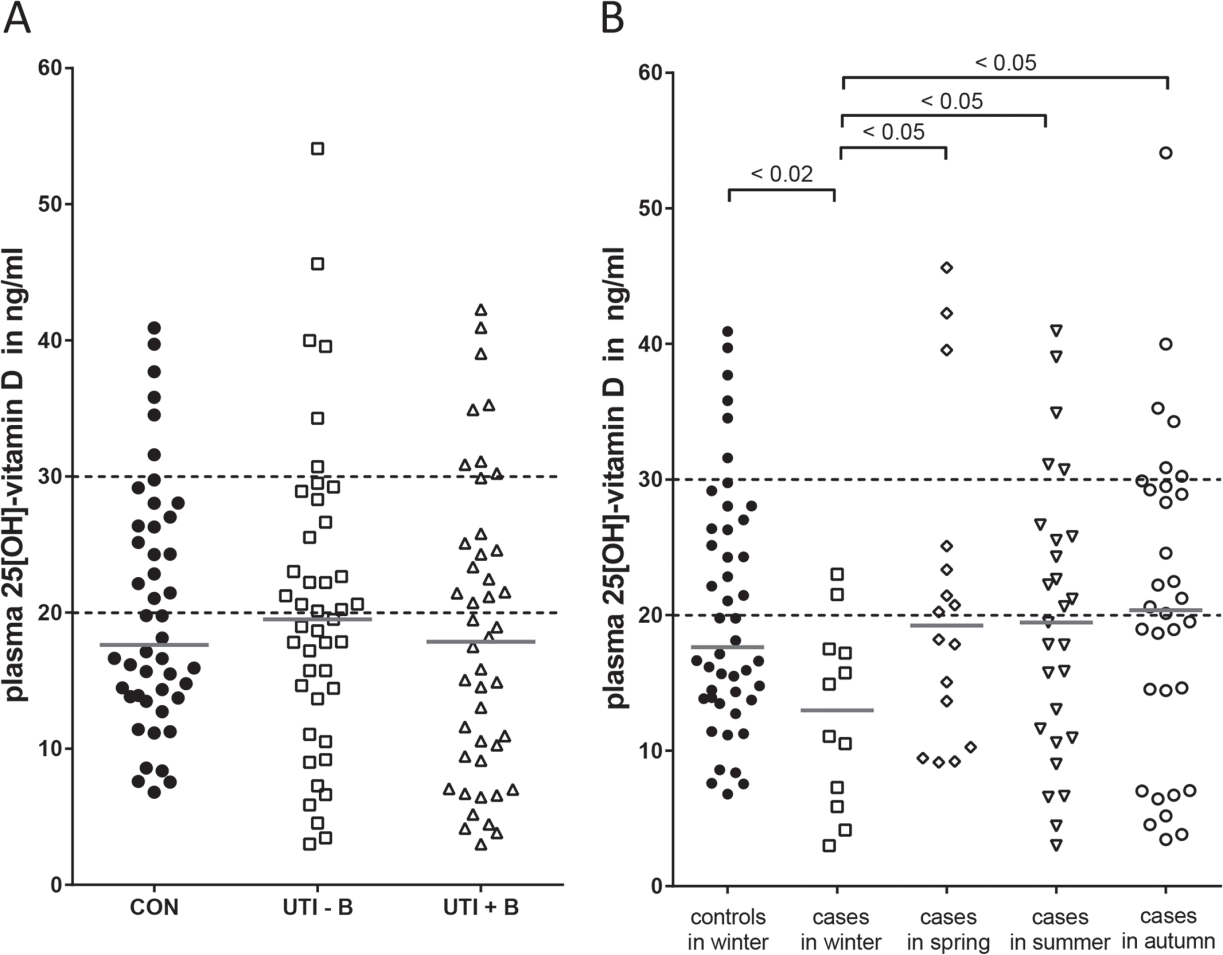
Plasma vitamin D concentrations in controls and UTI patients. Plasma 25(OH) vitamin D concentrations were determined in 46 controls (CON) and 43 UTI patients without bacteremia (UTI-B) and 44 UTI patients with bacteremia (UTI+B) (**A**). Analysis of plasma 25(OH)D vitamin D concentrations according to season of sampling (**B**). All controls were recruited in winter. 12 UTI patients were recruited in winter, 16 in spring, 27 in summer and 32 in autumn. Solid bars represent median concentrations; dotted lines represent upper limit of vitamin D deficiency (≤20 ng/ml) and lower limit of vitamin D sufficiency (≥30 ng/ml). The Mann-Whitney U test was used to determine whether groups were statistically different.

Plasma 25(OH)D was measured in 46 controls, 43 UTI patients without bacteremia and 44 UTI patients with bacteremia. All groups had comparable median plasma 25(OH)D concentrations (**Fig. 4A** and **Table 3**).

**Table 3:** Plasma 25(OH)D concentrations and vitamin D status.

	controls,	febrile UTI without	febrile UTI with
	*n* = 46	bacteremia, *n* = 43	bacteremia, *n* = 44
Median 25(OH)D in ng/ml [IQR]	17.6 [13.9–26.4]	19.5 [14.1–26.1]	17.9 [9.4–24.7]
vitamin D sufficient	6 (13%)	6 (14%)	8 (18%)
vitamin D insufficient	14 (30%)	15 (35%)	11 (25%)
vitamin D deficient	26 (57%)	22 (51%)	25 (57%)

UTI = urinary tract infection, IQR = interquartile range. Vitamin D sufficiency is defined as 25(OH)D of ≥30 ng/ml, vitamin D insufficiency is defined as 25(OH)D >20 and <30 ng/ml, vitamin D deficiency is defined as 25(OH)D of ≤ 20 ng/ml.

Of the 133 individuals analysed, only 22 (17%) had a sufficient vitamin D level. More than half (n = 73, 55%) had a vitamin D deficiency, while severe vitamin D deficiency (with 25(OH)D ≤10 ng/ml) was found in 25 participants (19%): 5 controls (11%), 8 UTI patients without bacteremia (19%) and 12 UTI patients with bacteremia (27%). Because vitamin D concentrations vary by season, we examined the effect of seasonal variation on our findings. All controls were recruited in winter. Patients with UTI were included in all seasons: 58 (44%) in winter, 16 (12%) in spring, 27 (20%) in summer and 32 (24%) in fall. The vitamin D concentration was significantly lower in UTI patients recruited in winter than in other seasons. More importantly, the controls that were all recruited in winter had a higher vitamin D concentration compared to cases recruited in winter (17.6 [IQR 13.9–26.4] ng/ml vs. 13.0 [IQR 6.9–17.3] ng/ml, p = 0.015) (**Fig. 4B**). Cathelicidin LL37 (r = -0.69, p = 0.436) and β-defensin 2 (r = -0.003, p = 0.969) in the urine were not correlated with plasma vitamin D.

### Distribution of genetic variation in cases and controls

In most infectious diseases, in addition to exposure, strain virulence and environmental factors, genetic host variations also play a role in susceptibility to disease. To determine whether genetic variations attribute to susceptibility to febrile UTI, we genotyped 15 single nucleotide polymorphisms (SNPs) in 12 genes of 440 controls and 707 febrile UTI patients (**Table 4**). All SNPs were in Hardy Weinberg equilibrium in the controls.

**Table 4:** Genotypes of genetic variations in controls and UTI patients.

		common name		controls	UTI patients	
Gene	SNPid	or location	genotypes	genotypes, n (%)	genotypes, n (%)	p-value
*CXCR1*	rs3138060	217C>G	CC	380 (87.6%)	623 (88.3%)	0.581
		in intron	CG	52 (12.0%)	82 (11.6%)	
			GG	2 (0.4%)	1 (0.1%)	
*CXCR1*	rs2234671	2608 G/C*	GG	379 (87.1%)	620 (88.0%)	0.574
		Ser276Thr	CG	54 (12.4%)	84 (11.9%)	
			CC	2 (0.5%)	1 (0.1%)	
*DEFA4*	rs28661751	A8P	CC	427 (98.4%)	700 (99.3%)	0.373
			CG	7 (1.6%)	6 (0.7%)	
			GG	0	0	
*DEFA4*	rs736227	in 3’UTR	TT	196 (45.4%)	324 (46.1%)	0.962
			CT	196 (45.4%)	313 (44.5%)	
			CC	40 (9.2%)	66 (9.4%)	
*DEFB1*	rs1800972	-44C>G*	CC	264 (60.8%)	426 (60.4%)	0.450
		-668C>G*	CG	147 (33.9%)	252 (35.7%)	
			GG	23 (5.3%)	27 (3.8%)	
*IL6*	rs10499563	-6331T>C*	TT	277 (63.8%)	403 (57.4%)	0.100
			CT	141 (32.5%)	269 (38.3%)	
			CC	16 (3.7%)	30 (4.3%)	
*IL6*	rs1800795	-174G>C*	GG	147 (33.7%)	249 (35.2%)	0.711
			CG	209 (47.9%)	340 (48.2%)	
			CC	80 (18.4)	117 (16.6%)	
*IL8*	rs4073	-251A/T*	TT	130 (29.9%)	207 (29.4%)	0.985
			AT	212 (48.7%)	346 (49.1%)	
			AA	93 (21.4%)	151 (21.5%)	
*MYD88*	rs6853	in 3’UTR	AA	331 (76.6%)	551 (78.6%)	0.120
			AG	92 (21.3%)	145 (20.7%)	
			GG	9 (2.1%)	5 (0.7%)	
*UMOD*	rs4293393	5’ near gene	TT	286 (65.9%)	460 (65.2%)	0.354
			CT	127 (29.3%)	220 (31.1%)	
			CC	21 (4.8%)	26 (3.7%)	
*TIRAP*	rs8177374	S180L*	CC	310 (71.1%)	498 (70.0%)	0.995
		C539T*	CT	116 (26.6%)	189 (26.7%)	
			TT	10 (2.3%)	16 (2.3%)	
*TLR1*	rs5743618	1805 G/T*	GG	179 (42.0%)	315 (45.4%)	0.379
		S602I*	GT	193 (45.3%)	285 (41.1%)	
			TT	54 (12.7%)	94 (13.5%)	
*TLR2*	rs5743708	Arg753Gln*	GG	408 (93.2%)	662 (93.8%)	0.773
		R753Q*	AG	30 (6.8%)	44 (6.2%)	
			AA	0	0	
*TLR5*	rs5744168	R392X*	CC	363 (84.2%)	626 (89.0%)	0.072
		Arg392Stop*	CT	63 (14.6%)	72 (10.2%)	
		1174C>T*	TT	5 (1.2%)	6 (0.8%)	
*TNF*	rs1800629	-308 G/A*	GG	282 (64.6%)	486 (68.8%)	0.170
		in promoter	AG	144 (33.0%)	197 (27.9%)	
			AA	11 (2.5%)	23 (3.3%)	

* name used in the literature

None of the SNP alleles (data not shown) or genotypes were found to be associated with susceptibility to febrile UTI when analyzing controls versus patients (**Table 4**). Because the presence of urinary tract disorders or chronic renal insufficiency may mask genetic effects we re-analyzed the data after exclusion of individuals with known urinary tract disorders or chronic renal insufficiency. This revealed that the *TLR5* SNP rs5744168 was significantly different between patients and controls (p = 0.011). In addition we analyzed the distribution of genotypes between UTI patients with and those without bacteremia. The distribution of one SNP, rs1800795 in the gene *IL6*, was significantly different as the genotype CC is more common among UTI patients with bacteremia (p = 0.009) (**Table 5**).

**Table 5:** Genotypes of *IL6* variation in UTI patients with and without bacteremia.

			UTI without	UTI with	p-value
Gene	SNPid	genotypes	bacteremia n (%)	bacteremia n (%)	
*IL6*	rs1800795	CC	76 (15.0%)	37 (23.9%)	0.009
		CG	257 (50.9%)	57 (36.8%)	
		GG	172 (34.1%)	61 (39.3%)	

**Fig 5:**
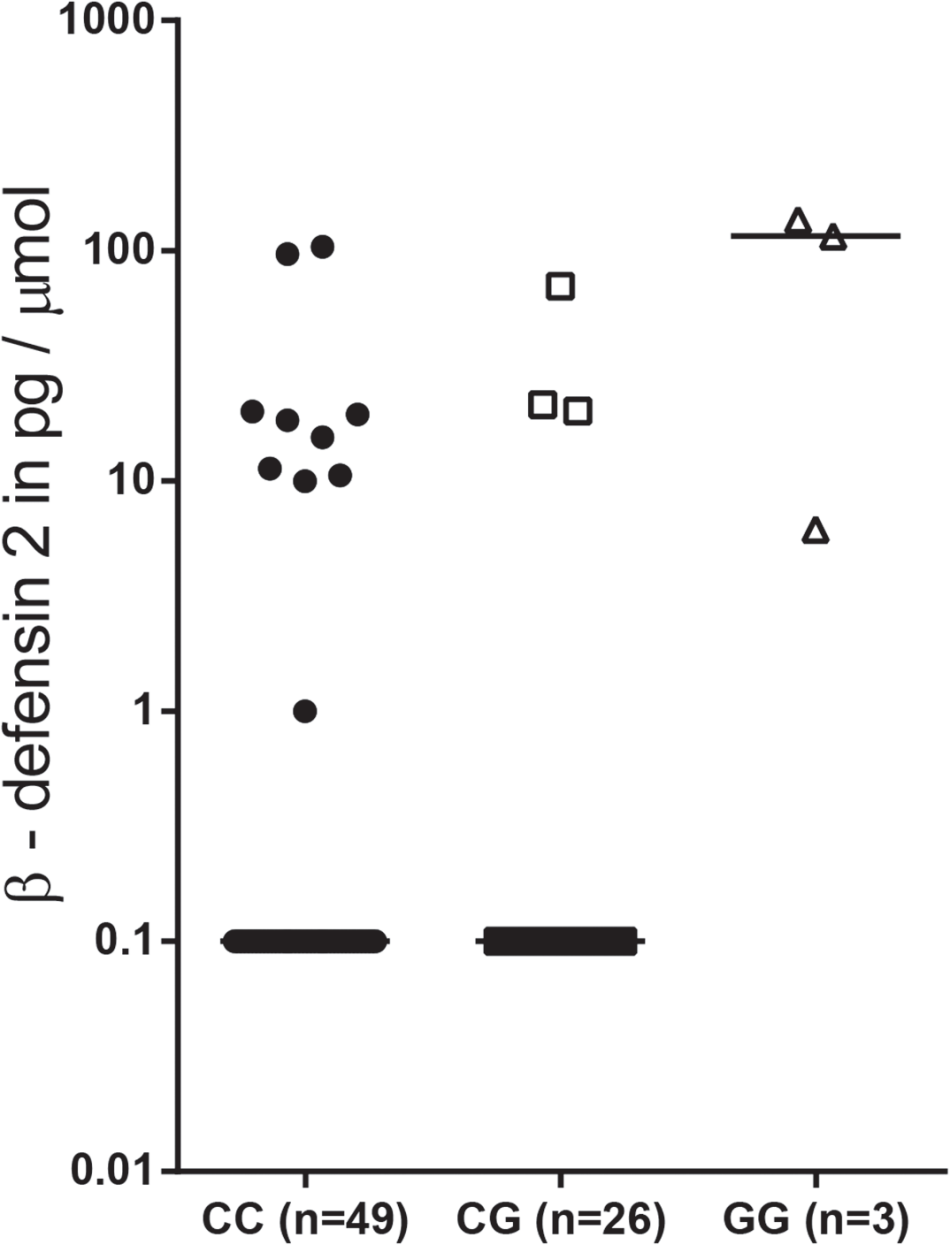
β-defensin 2 production is associated with a DEFB1 genotype. Concentration of the antimicrobial protein β-defensin 2 grouped per DEFB1 rs1800972 genotype, the groups are significantly different (p = 0.0002). Protein concentrations were corrected for urine gravity, for samples below the detection limit the corrected cytokine concentration was set to 0.1. Solid bars represent medians, for statistical analysis the Kruskal-Wallis test was used.

The production of proteins can often be correlated to genetic variations in for instance the promoter region or translation start site. Most individuals in which urinary protein concentrations were measured in this study were also genotyped, therefore we were able to investigate whether any of the genotypes were correlated to urinary protein production. A significant difference in β-defensin 2 production was found between the *DEFB1* rs1800972 genotypes, as most individuals with an CC or CG genotype did not produce β-defensin 2 while the three individuals with an GG genotype did (p = 0.0002) (**Fig. 5**). No significant correlations were found between other genotypes and protein production (data not shown).

## Discussion

We analyzed various proteins in urine of UTI patients and controls from a prospective cohort study and found that while IL-6, IL-8 and cathelicidin LL37 are different between UTI patients and controls, these proteins, as well as β-defensin 2, cannot be used as biomarkers for bacteremia. We did find that while uromodulin production is in general not different between patients and controls, complete absence of uromodulin production in the urine is a strong risk factor for bacteremia. In addition we found that plasma vitamin D may have a protective effect against UTI as, at least in winter, vitamin D is lower in UTI patients compared to controls.

The strength of this study is that we have collected the largest prospective UTI cohort thus far with a clinically and microbiologically well-characterized disease group. Our emphasis in the analyses was on the identification of host defense risk factors for febrile UTI and predictive markers for bacteremia. Therefore we determined urinary proteins and plasma vitamin D in 91 patients (of which 46 had bacteremia) and 46 controls, and genotyped polymorphisms in genes thought to play a role in UTI in a large number of patients (n = 707) and controls (n = 440). The three groups of individuals in which urinary proteins and plasma vitamin D were determined were in general well matched, except for age which was higher in patients with UTI and bacteremia.

In general, IL-6 and IL-8 levels are elevated in the urine of patients with UTI and children with acute pyelonephritis, whereas none are measurable in the urine of controls [6–10]. This study confirms that IL-6 and IL-8 are elevated in UTI patients; we did however also find four controls that produced IL-8. One of the controls had asymptomatic bacteriuria; for the other three it is unclear why they produce IL-8. Production of IL-8 has also occasionally been observed in healthy children [8,9]. While IL-6 and IL-8 are good biomarkers for infection, we found that they cannot distinguish between patients with or without bacteremia. In a Swedish study a significant difference in IL-6 production was found between bacteremic and non-bacteremic patients, 24 hours after inclusion [32]. In that study, UTI patients were all hospitalized, suggesting they had far more severe clinical symptoms than our patients who were largely recruited at family practices. In addition, similar to the samples in our cohort, at inclusion IL-6 production in the Swedish patients was not significantly different between patients with or without bacteremia [32].

As previously reported in children and adults with UTI [11,13], we also found that urinary cathelicidin LL37 is elevated in adults with UTI and appears to be a biomarker for infection. Cathelicidin LL37 was however not different between patients with and without bacteremia, making it unsuitable as a biomarker for bacteremia.

β-defensin 2 has been previously shown by RT-PCR and immunohistochemistry to be produced in kidneys from patients with chronic upper UTI undergoing nephrectomy but not in normal kidneys [12]. In addition it was reported that β-defensin 2 mRNA transcription by renal tubular cells *in vitro* could be upregulated by *E*. *coli* [33]. We determined the β-defensin 2 protein production by ELISA and found that β-defensin 2 was detectable in the urine of a minority of both controls and patients and did not differ between them. There was also no difference in β-defensin 2 production between patients with or without bacteremia, making it unsuitable as a biomarker for bacteremia.

Nitschke *et al* observed that stimulation with IL-6 did not affect β-defensin 2 production in renal tubular cells [33].We determined whether there was a relation between IL-6 and β-defensin 2 in urine. 30 out of 89 patients produce IL-6 and 23 out of 89 patients produce β-defensin 2 while only six produce both, which is even slightly less than the expected number (eight) if they are not correlated. This suggests that these two proteins are not induced by the same stimuli.

Uromodulin is known to be produced constitutively in urine and was previously reported not to be correlated with recurrent UTI in young women [15].We found that the production also did not differ between the elderly patients and controls in our cohort or between patients with or without bacteremia. We did however observe that the inability to produce uromodulin in the urine greatly increased the risk of developing bacteremia (OR 6.0, 95% CI: 1.2–29.2). Uromodulin is produced in the thick ascending loop of Henle and secreted into the urine via proteolytic cleavage. It is a known biomarker for kidney disease [34]. Of the 16 individuals who did not produce detectable uromodulin only a few had a condition that affected the kidneys: two patients with bacteremia had chronic kidney insufficiency and one other had urothelial cell carcinoma. One control had only one kidney but that in itself does not explain the lack of uromodulin production. Lack of uromodulin production in the urine appears to be a valuable biomarker for bacteremia in vulnerable UTI patients.

The majority of the individuals in our study had, according to commonly used standards [31], insufficient vitamin D in their blood, while severe vitamin D deficiency was found in many as well. However, there is still debate about the definition of vitamin deficiency [35] but regardless of the criteria used we found comparable median plasma vitamin D concentrations between the three groups. Unfortunately, all samples for vitamin D measurement in controls were recruited in winter. That may mask a difference between UTI patients and controls. When comparing controls with patients recruited in the same season, controls had significantly higher plasma vitamin D. Previously, low vitamin D levels were shown to be associated with bacterial and viral infections [36]. A recent study also showed that vitamin D levels in patients with recurrent UTI were found to be lower than in controls [20]. However, in that study it was unclear whether recruitment of cases and controls were equally distributed upon the different seasons. Based on *in vitro* experiments with bladder biopsies from women before and after vitamin D supplementation it has been suggested that the protective effect of vitamin D against UTI may be through the regulation of cathelicidin LL37 production in the bladder [19]. In this respect, one might expect that higher vitamin D levels might to some extent have a protective effect against invasive UTI and bacteremia via cathelicidin. We however did not find an association of plasma vitamin D with urinary cathelicidin LL37. Furthermore, there was no association between either vitamin D level or urinary cathelicidin LL37 with the presence of bacteremia.

We selected fifteen polymorphisms in genes with a known role in the recognition, the defense, or immune response to uropathogens (*TLR1*, *TLR2*, *TLR5*, *TIRAP*, *MYD88*, *IL6*, *IL8*, *DEFA4*, *DEFB1*, *UMOD*, *CXCR1*, *TNF*). None of the alleles or genotypes was associated with febrile UTI when analyzing all controls versus all patients. Because the presence of urinary tract disorders or chronic renal insufficiency may mask genetic effects we re-analysed these data after exclusion of individuals with known urinary tract disorders or chronic renal insufficiency. This revealed an association between *TLR5* SNP rs5744168 and *protection from* UTI. The same SNP was previously found to be associated with *increased susceptibilit*y to recurrent UTI in Caucasian American women (339 recurrent UTI, 321 pyelonephritis, 317 controls) [26]. The discrepancy in the allele associated with protection from UTI suggests that the *TLR5* SNP is not itself the functional variant causing the protective effect but rather linked to a functional variation in the vicinity. The different genetic backgrounds in the two studies may cause the functional variant to co-segregate with different alleles. The *TLR5* polymorphism is also a lot more common in our population than in the Caucasian American population (CT and TT 15.8% in our controls vs 7.4% in the Caucasian American controls) thus revealing a difference in distribution. More SNPs in and around *TLR5* need to be analyzed to pinpoint the causal variation.

Various other genetic variations have also been reported to be associated with protection from, or the risk of developing UTI [27]. We analyzed several of these but none were associated with febrile UTI in our cohort: the *TLR2* polymorphism Arg753Gln that was associated with UTI in Turkish children (124 patients, 116 controls) [24], the *TLR1* 1805T allele that was associated with pyelonephritis in Caucasian American women (339 recurrent UTI, 321 pyelonephritis, 317 controls) [26] and five *CXCR1* polymorphisms (of which we tested two) that were associated with acute pyelonephritis in a Swedish cohort (60 patients, 226 controls) [37]. These associations have thus far not been confirmed in other independent cohorts either. The lack of association in our cohort may be due to a difference in genetic background or due to the selection of elderly patients in our cohort, as at an elderly age comorbidity and an aging immune system may have more effect on susceptibility to UTI than subtle genetic variations. We analyzed the largest cohort so far (440 controls and 707 patients) therefore the power to detect an association (if present) is greater than in the other cohorts.

We found one polymorphism, the *IL6* SNP rs1800795, specifically the CC genotype, to be associated with development of bacteremia. This SNP is located in the promoter of *IL6* and is known to affect the IL-6 concentration in blood plasma, with the GG genotype producing more IL-6 [38,39]. Unfortunately, because not enough individuals produce IL-6 in their urine we were unable to determine whether the GG genotype is also correlated with IL-6 production in urine. It is however likely that the CC genotype increases susceptibility to UTI due to low IL-6 production. In one study, a significantly higher urinary IL-6 production was found in bacteremic than in non-bacteremic patients, at 24 hr after inclusion [32]. In that study it was also found that IL-6 response kinetics were different in bacteremic and non-bacteremic patients, with IL-6 in non-bacteremic patients peaking early and declining afterwards while IL-6 in bacteremic patients peaks late and stays high [32]. It would be interesting to determine whether these response kinetics are under genetic control. In mouse models of bladder infection with uropathogenic *E*. *coli*, transcription of the *il6* gene was found to be very highly upregulated at 2 hr and moderately upregulated at 24 hr after infection, with response kinetics varying between mouse strains, suggesting genetic control [40]. In addition, the causative uropathogen was also found to influence the response kinetics, with group B streptococcus inducing a far less pronounced response than *E*. *coli* [41].

In conclusion, we did not identify a convenient biomarker that will allow us to distinguish between patients with or without bacteremia. We did find a risk factor, lack of uromodulin, that is present in few patients but greatly increases their risk of developing bacteremia. In addition we identified several modest genetic associations, especially between *TLR5* SNP rs5744168 and UTI and between *IL6* SNP rs1800795 and occurrence of bacteremia.

## References

[1] Ulett GC, Totsika M, Schaale K, Carey AJ, Sweet MJ, Schembri MA. Uropathogenic Escherichia coli virulence and innate immune responses during urinary tract infection. Curr Opin Microbiol. 2013;16: 100–107. 10.1016/j.mib.2013.01.005 23403118

[2] Kunin CM. Definition of acute pyelonephritis vs the urosepsis syndrome. Arch Intern Med. 2003;163: 2393 14581259 10.1001/archinte.163.19.2393-b

[3] van Nieuwkoop C, van't Wout JW, Spelt IC, Becker M, Kuijper EJ, Blom JW, et al Prospective cohort study of acute pyelonephritis in adults: Safety of triage towards home based oral antimicrobial treatment. J Infect. 2010;60: 114–121. 10.1016/j.jinf.2009.11.008 19945482

[4] van Nieuwkoop C, Bonten TN, van't Wout JW, Kuijper EJ, Groeneveld GH, Becker MJ, et al Procalcitonin reflects bacteremia and bacterial load in urosepsis syndrome: a prospective observational study. Crit Care. 2010;14: R206 10.1186/cc9328 21083886 PMC3220019

[5] van Nieuwkoop C, Bonten TN, van't Wout JW, Becker MJ, Groeneveld GH, Jansen CL, et al Risk Factors for Bacteremia with Uropathogen Not Cultured from Urine in Adults with Febrile Urinary Tract Infection. Clin Infect Dis. 2010;50: e69–e72. 10.1086/652657 20420504

[6] Hedges S, Stenqvist K, Lidin-Janson G, Martinell J, Sandberg T, Svanborg C. Comparison of urine and serum concentrations of Interleukin-6 in women with acute pyelonephritis or asymptomatic bacteriuria. J Infect Dis. 1992;166: 653–656. 1500753 10.1093/infdis/166.3.653

[7] Ko YC, Mukaida N, Ishiyama S, Tokue A, Kawai T, Matsushima K, et al Elevated interleukin-8 levels in the urine of patients with urinary tract infections. Infect Immun. 1993;61: 1307–1314. 8454332 10.1128/iai.61.4.1307-1314.1993 PMC281363

[8] Tullus K, Fituri O, Burman LG, Wretlind B, Brauner A. Interleukin-6 and interleukin-8 in the urine of children with acute pyelonephritis. Pediatr Nephrol. 1994;8: 280–284. 7917851 10.1007/BF00866334

[9] Sheu JN, Chen MC, Lue KH, Cheng SL, Lee IC, Chen SM, et al Serum and urine levels of interleukin-6 and interleukin-8 in children with acute pyelonephritis. Cytokine. 2006;36: 276–282. 17374489 10.1016/j.cyto.2007.02.006

[10] Renata Y, Jassar H, Katz R, Hochberg A, Nir RR, Klein-Kremer A. Urinary concentration of cytokines in children with acute pyelonephritis. Eur J Pediatr. 2013;172: 769–774. 10.1007/s00431-012-1914-2 23389820

[11] Chromek M, Slamova Z, Bergman P, Kovacs L, Podracka L, Ehren I, et al The antimicrobial peptide cathelicidin protects the urinary tract against invasive bacterial infection. Nat Med. 2006;12: 636–641. 16751768 10.1038/nm1407

[12] Lehmann J, Retz M, Harder J, Krams M, Kellner U, Hartmann J, et al Expression of human beta-defensins 1 and 2 in kidneys with chronic bacterial infection. BMC Infect Dis. 2002;2: 20 12238953 10.1186/1471-2334-2-20 PMC128826

[13] Nielsen KL, Dynesen P, Larsen P, Jakobsen L, Andersen PS, Frimodt-Møller N. The Role of Urinary Cathelicidin (LL-37) and Human β-defensin 1 (hBD-1) in Uncomplicated Escherichia coli Urinary Tract Infections. Infect Immun. 2014;82: 1572–1578. 10.1128/IAI.01393-13 24452682 PMC3993379

[14] Chromek M, Brauner A. Antimicrobial mechanisms of the urinary tract. J Mol Med. 2008;86: 37–47. 17805504 10.1007/s00109-007-0256-4

[15] Reinhart H, Obedeanu N, Hooton T, Stamm W, Sobel J. Urinary excretion of Tamm-Horsfall protein in women with recurrent urinary tract infections. J Urol. 1990;144: 1185–1187. 1977928 10.1016/s0022-5347(17)39687-8

[16] Visser M, Deeg DJH, Lips P. Low vitamin D and high parathyroid hormone levels as determinants of loss of muscle strength and muscle mass (sarcopenia): the longitudinal aging study Amsterdam. J Clin Endocrinol Metab. 2003;88: 5766–5772. 14671166 10.1210/jc.2003-030604

[17] Snijder MB, van Dam RM, Visser M, Deeg DJ, Dekker JM, Bouter LM, et al Adiposity in relation to vitamin D status and parathyroid hormone levels: a population-based study in older men and women. J Clin Endocrinol Metab. 2005;90: 4119–4123. 15855256 10.1210/jc.2005-0216

[18] Lips P. Worldwide status of vitamin D nutrition. J Steroid Biochem Mol Biol. 2010;121: 297–300. 10.1016/j.jsbmb.2010.02.021 20197091

[19] Hertting O, Holm A, Luthje P, Brauner H, Dyrdak R, Jonasson AF, et al Vitamin D induction of the human antimicrobial Peptide cathelicidin in the urinary bladder. PLoS One. 2010;5: e15580 10.1371/journal.pone.0015580 21179490 PMC3001888

[20] Nseir W, Taha M, Nemarny H, Mograbi J. The association between serum levels of vitamin D and recurrent urinary tract infections in premenopausal women. Int J Infect Dis. 2013;17: e1121–e1124. 10.1016/j.ijid.2013.06.007 23911156

[21] Cooke GS, Hill AV. Genetics of susceptibility to human infectious disease. Nat Rev Genet. 2001;2: 967–977. 11733749 10.1038/35103577

[22] Casanova JL, Abel L. The human model: a genetic dissection of immunity to infection in natural conditions. Nat Rev Immunol. 2004;4: 55–66. 14704768 10.1038/nri1264

[23] Scholes D, Hawn TR, Roberts PL, Li SS, Stapleton AE, Zhao LP, et al Family history and risk of recurrent cystitis and pyelonephritis in women. J Urol. 2010;184: 564–569. 10.1016/j.juro.2010.03.139 20639019 PMC3665335

[24] Tabel Y, Berdeli A, Mir S. Association of TLR2 gene Arg753Gln polymorphism with urinary tract infection in children. Int J immunogenet. 2007;34: 399–405. 18001294 10.1111/j.1744-313X.2007.00709.x

[25] Karoly E, Fekete A, Banki NF, Szebeni B, Vannay A, Szabo AJ, et al Heat Shock Protein 72 (HSPA1B) gene polymorphism and Toll-Like Receptor (TLR) 4 mutation are associated with increased risk of urinary tract infection in children. Pediatr Res. 2007;61: 371–374. 17314700 10.1203/pdr.0b013e318030d1f4

[26] Hawn TR, Scholes D, Li SS, Wang H, Yang Y, Roberts PL, et al Toll-Like receptor polymorphisms and susceptibility to urinary tract infections in adult women. PLoS ONE. 2009;4: e5990 10.1371/journal.pone.0005990 19543401 PMC2696082

[27] Ragnarsdottir B, Lutay N, Gronberg-Hernandez J, Koves B, Svanborg C. Genetics of innate immunity and UTI susceptibility. Nat Rev Urol. 2011;8: 449–468. 10.1038/nrurol.2011.100 21750501

[28] Aslan S, Akil I, Aslan G, Onay H, Ozyurt BC, Ozkinay F. Vitamin D receptor gene polymorphism in children with urinary tract infection. Pediatr Nephrol. 2012;27: 417–421. 10.1007/s00467-011-2000-0 21947233

[29] Javor J, Kralinsky K, Sadova E, Cervenova O, Bucova M, Olejarova M, et al Association of interleukin-10 gene promoter polymorphisms with susceptibility to acute pyelonephritis in children. Folia Microbiol. 2014;59: 307–313. 10.1007/s12223-014-0303-9 24449078

[30] Sambrook J, Russell DW. Molecular cloning: a laboratory manual Cold Spring Harbor, New York: Cold Spring Harbor Laboratory Press; 2001.

[31] Holick MF. Vitamin D Deficiency. New Engl J Med. 2007;357: 266–281. 17634462 10.1056/NEJMra070553

[32] Otto G, Braconier J, Andreasson A, Svanborg C. Interleukin-6 and Disease Severity in Patients with Bacteremic and Nonbacteremic Febrile Urinary Tract Infection. J Infect Dis. 1999;179: 172–179. 9841836 10.1086/314534

[33] Nitschke M, Wiehl S, Baer PC, Kreft B. Bactericidal activity of renal tubular cells: the putative role of human beta-defensins. Exp Nephrol. 2002;10: 332–337. 12381917 10.1159/000065296

[34] Rampoldi L, Scolari F, Amoroso A, Ghiggeri G, Devuyst O. The rediscovery of uromodulin (Tamm-Horsfall protein): from tubulointerstitial nephropathy to chronic kidney disease. Kidney Int. 2011;80: 338–347. 10.1038/ki.2011.134 21654721

[35] Rosen CJ. Vitamin D Insufficiency. N Engl J Med. 2011;364: 248–254. 10.1056/NEJMcp1009570 21247315

[36] Borella E, Nesher G, Israeli E, Shoenfeld Y. Vitamin D: a new anti-infective agent? Ann NY Acad Sci. 2014;1317: 76–83. 10.1111/nyas.12321 24593793

[37] Lundstedt AC, McCarthy S, Gustafsson MCU, Godaly G, Jodal U, Karpman D, et al A Genetic Basis of Susceptibility to Acute Pyelonephritis. PLoS ONE. 2007;2: e825 17786197 10.1371/journal.pone.0000825 PMC1950574

[38] Fishman D, Faulds G, Jeffery R, Mohamed-Ali V, Yudkin JS, Humphries S, et al The effect of novel polymorphisms in the interleukin-6 (IL-6) gene on IL-6 transcription and plasma IL-6 levels, and an association with systemic-onset juvenile chronic arthritis. J Clin Invest. 1998;102: 1369–1376. 9769329 10.1172/JCI2629 PMC508984

[39] Zakharyan R, Petrek M, Arakelyan A, Mrazek F, Atshemyan S, Boyajyan A. Interleukin-6 promoter polymorphism and plasma levels in patients with schizophrenia. Tissue Antigens. 2012;80: 136–142. 10.1111/j.1399-0039.2012.01886.x 22571276

[40] Duell BL, Carey AJ, Tan CK, Cui X, Webb RI, Totsika M, et al Innate transcriptional networks activated in bladder in response to uropathogenic *Escherichia coli* drive diverse bological pathways and rapid synthesis of IL-10 for defense against bacterial urinary tract infection. J Immunol. 2012;188: 781–792. 10.4049/jimmunol.1101231 22184725

[41] Tan CK, Carey AJ, Cui X, Webb RI, Ipe D, Crowley M, et al Genome-wide mapping of cystitis due to *Streptococcus agalactiae* and *Escherichia coli* in mice identifies a unique bladder transcriptome that signifies pathogen-specific antimicrobial defense against urinary tract infection. Infect Immun. 2012;80: 3145–3160. 10.1128/IAI.00023-12 22733575 PMC3418756

